# Gamma Delta T-Cell Based Cancer Immunotherapy: Past-Present-Future

**DOI:** 10.3389/fimmu.2022.915837

**Published:** 2022-06-16

**Authors:** José Saura-Esteller, Milon de Jong, Lisa A. King, Erik Ensing, Benjamin Winograd, Tanja D. de Gruijl, Paul W. H. I. Parren, Hans J. van der Vliet

**Affiliations:** ^1^ Department of Medical Oncology, Cancer Center Amsterdam, Amsterdam Infection and Immunity Institute, Amsterdam University Medical Center (UMC), Vrije Universiteit Amsterdam, Amsterdam, Netherlands; ^2^ LAVA Therapeutics, Utrecht, Netherlands; ^3^ LAVA Therapeutics, Philadelphia, PA, United States; ^4^ Department of Immunology, Leiden University Medical Center, Leiden, Netherlands

**Keywords:** gamma delta T-cell, cancer, immunotherapy, phosphoantigens, aminobisphosphonates, adoptive cell transfer, bispecific t-cell engager, chimeric antigen receptor

## Abstract

γδ T-cells directly recognize and kill transformed cells independently of HLA-antigen presentation, which makes them a highly promising effector cell compartment for cancer immunotherapy. Novel γδ T-cell-based immunotherapies, primarily focusing on the two major γδ T-cell subtypes that infiltrate tumors (*i.e.* Vδ1 and Vδ2), are being developed. The Vδ1 T-cell subset is enriched in tissues and contains both effector T-cells as well as regulatory T-cells with tumor-promoting potential. Vδ2 T-cells, in contrast, are enriched in circulation and consist of a large, relatively homogeneous, pro-inflammatory effector T-cell subset. Healthy individuals typically harbor in the order of 50-500 million Vγ9Vδ2 T-cells in the peripheral blood alone (1-10% of the total CD3^+^ T-cell population), which can rapidly expand upon stimulation. The Vγ9Vδ2 T-cell receptor senses intracellular phosphorylated metabolites, which accumulate in cancer cells as a result of mevalonate pathway dysregulation or upon pharmaceutical intervention. Early clinical studies investigating the therapeutic potential of Vγ9Vδ2 T-cells were based on either *ex vivo* expansion and adoptive transfer or their systemic activation with aminobisphosphonates or synthetic phosphoantigens, either alone or combined with low dose IL-2. Immune-related adverse events (irAE) were generally \mild, but the clinical efficacy of these approaches provided overall limited benefit. In recent years, critical advances have renewed the excitement for the potential of Vγ9Vδ2 T-cells in cancer immunotherapy. Here, we review γδ T-cell-based therapeutic strategies and discuss the prospects of those currently evaluated in clinical studies in cancer patients as well as future therapies that might arise from current promising pre-clinical results.

## Introduction

**Figure 1:**
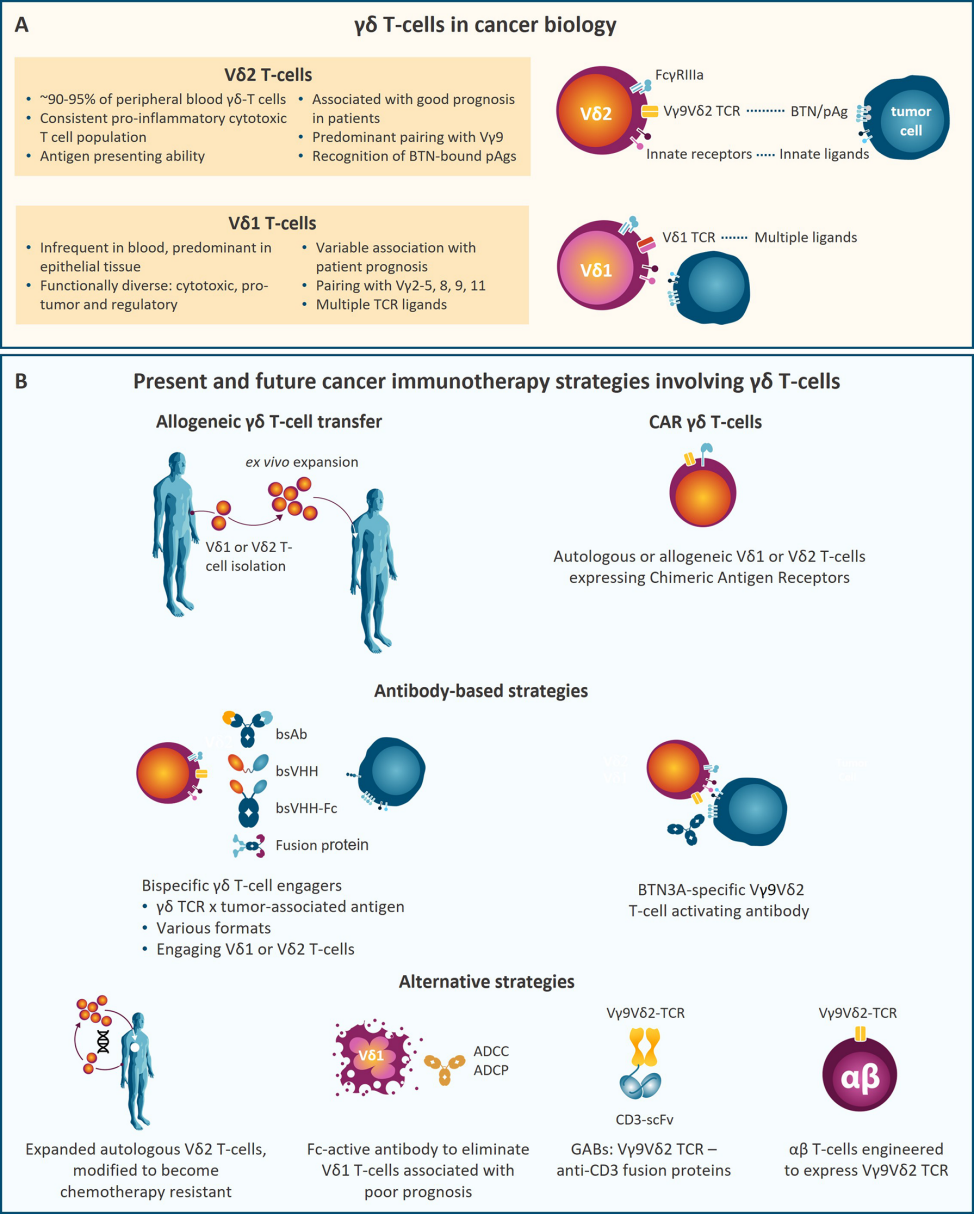
**(A)** Key characteristics of the two main γδ T-cell subsets, Vδ2 and Vδ1 T-cells, in cancer biology. **(B)** Schematic representation of therapeutic strategies involving γδ T-cells that are currently being developed. ADCC, Antibody-dependent cellular cytotoxicity; ADCP, antibody-dependent cellular phagocytosis; bsAb, bispecific antibody; bsVHH, bispecific variable domain of heavy-chain only antibody; BTN, Butyrophilin; CAR, chimeric antigen receptor; pAg, phosphoantigen; scFv, single-chain variable fragment.

In humans, γδ T-cells represent 1 to 10% of total CD3^+^ T-cells (1, 2), and express a combination of either of 7 different Vγ TCR chains (Vγ2, 3, 4, 5, 8, 9, and 11), paired with either of 4 Vδ (Vδ1, 2, 3, and 5) chains (2–4). γδ T-cells are considered to bridge the innate and adaptive immune systems (3). Activated γδ T-cells display strong cytotoxic activity through the release of granzyme B and perforin, by membrane bound TRAIL and Fas (CD95) ligands or production of IFNγ or TNFα to amplify the immune response (12), thereby counteracting tumor development. Using γδ T-cell-deficient mice in a cutaneous carcinogenesis model, γδ T-cells were first shown to prevent malignancy formation (5). High γδ T-cell frequency in tumor infiltrates from cancer patients correlates with better clinical outcome in different malignancies (6–10) and γδ T-cells were identified as the prognostically most favorable immune cell subset in tumor infiltrates from 18,000 tumors across 39 malignancies (11). A more recent study confirmed the relative abundance of Vγ9Vδ2 T-cells in TILs and their association with improved patient outcome (12). These results highlight the relevance of γδ T-cells in tumor control and their potential for cancer therapy. γδ T-cells express several receptors shared with natural killer (NK) cells that participate in enhanced tumor cell recognition of which FcγRIIIa (CD16a), DNAM-1, and NKG2D are a few examples (13) (**Figure 1A**).

The complete repertoire of antigens recognized by γδ-TCRs and the specificity of each γδ T subset is still not fully understood. Vγ9Vδ2 T-cells represent the predominant γδ T-cell subset (95%) in peripheral blood (14). Vγ9Vδ2 T-cells participate in the defense against malignant cells by sensing small phosphorylated metabolites (phosphoantigen (pAg) molecules) produced in cholesterol synthesis [isopentenyl pyrophosphate (IPP)] or by pathogens [*e.g*. (*E*)-4-hydroxy-3-methyl-but-2-enyl-pyrophosphate (HMBPP)] (5, 15–19). Unlike conventional αβ T-cells, ligand recognition by Vγ9Vδ2 and most γδ T-cells does not involve antigen presentation by human leukocyte antigen (HLA) molecules (15, 20). Ligand recognition by Vγ9Vδ2 T-cells requires butyrophilin (BTN) 3A1 (21) and BTN2A1 (22–24). Intracellular pAg levels are increased under stress conditions like infection or malignant transformation or by aminobisphosphonates (ABP) (16, 17, 25–27). Vγ9Vδ2 T-cells sense increased intracellular pAg levels causing their activation and target cell killing. Recent studies show that pAg-bound BTN3A1 associates with BTN2A1 which directly interacts with non-variable regions of the Vγ9 chain on γδ T-cells. Besides Vγ9Vδ2 T-cell recognition of pAgs, some subsets of Vδ1 and Vδ3 T-cells detect pathogenic and self-lipids presented by CD1d through their TCR (28, 29). Vδ1 T-cells are less abundant in circulation than Vγ9Vδ2 T-cells, but they are enriched in epithelia (30) and among tumor infiltrating lymphocytes (TILs). While cultured Vδ1 T-cells may have higher cytotoxic capacity than Vγ9Vδ2 T-cells, Vδ1 T-cells can be pro-tumoral in certain malignancies (6, 31, 32) (**Figure 1A**).

In this review we discuss γδ T-cell-based therapeutic strategies with a focus on recent developments of bispecific γδ T-cell engagers (bsTCEs) and chimeric antigen receptor (CAR) γδ T-cells, and point towards approaches that may develop into therapies in the near future (**Figure 1B**).

## Past Clinical Studies With Vγ9Vδ2 T-Cells

In the year 2000, ABP drugs, already approved to treat patients with excessive bone resorption, were shown to cause systemic Vγ9Vδ2 T-cell stimulation and to increase their antitumor activity in a preclinical study (26). Following this observation, studies explored ABP treatment as a systemic γδ T-cell stimulant or as an *ex vivo* tool to expand them for subsequent adoptive cell transfer (ACT) for cancer immunotherapy.

The ABPs pamidronate (PAM) and zoledronate (ZOL), and synthetic pAg analogues, mainly bromohydrin pyrophosphate (BrHPP) and 2-methyl-3-butenyl-1-pyrophosphate (2M3B1PP), have been used alone or in combination with IL-2 to activate Vγ9Vδ2 T-cells (33, 34). ABP treatment has been evaluated in cancer patients (*e.g.* with multiple myeloma (MM), non-Hodgkin lymphoma (NHL), acute myeloid leukemia (AML), prostate cancer, renal cell carcinoma, colorectal cancer, breast cancer, melanoma or neuroblastoma) (33, 35–39). Additionally, *ex vivo* expansion of autologous γδ T-cells with ABPs or synthetic pAg followed by ACT has been tested in a wide range of malignancies (*e.g.* in MM, renal cell carcinoma, non-small cell lung cancer, gastric cancer, hepatocellular carcinoma, melanoma, ovarian cancer, colon cancer and pancreatic cancer) (40–51). While these approaches were well tolerated, clinical responses typically were found to be infrequent and not long-lasting, though sporadic meaningful responses were achieved (52–54). The overall moderate clinical antitumor effect of systemic γδ T-cell activation with ABP or synthetic pAg and of autologous γδ T transfer, negatively impacted further development of these Vγ9Vδ2 T-cell-directed cancer immunotherapeutic approaches.

## Present and Future Studies Involving γδ T-Cells

### γδ T-Cell-Based Cellular Strategies

#### Allogeneic γδ T-Cell Transfer

As mentioned above, most γδ T-cells recognize target cells independently of HLA antigen presentation, suggesting that allogeneic donor derived γδ T-cells can be relatively safe for ACT due to low risk of graft-versus-host disease (GvHD). Taking advantage of this, current strategies exploring the use of *ex vivo* expanded γδ T-cell infusion have shifted towards allogeneic origin (**Table 1**). Increased frequency of γδ T-cells in leukemia patients that underwent αβ-depleted allogeneic stem cell transplantation from partially HLA-mismatched donors, was associated with a higher 5-year and overall survival (OS) (55, 56). A single infusion of allogeneic Vγ9Vδ2 T-cells, expanded *ex vivo* with ZOL plus IL-2, is being administered in a clinical trial (NCT03533816) to maximize antitumor response and reduce GvHD, after allogeneic hematopoietic cell transplant (alloHCT) and cyclophosphamide for hematologic malignancies. Moreover, allogeneic Vγ9Vδ2 T-cell infusion after lymphodepletion is being tested independently of alloHCT for hematologic malignancies and solid tumors. Some of these studies have already been completed with no major adverse effects reported, highlighting the safety of Vγ9Vδ2 T-cell transfer (57, 58). Importantly, patients receiving Vγ9Vδ2 T-cell infusion had increased OS compared to control patients and repeated Vγ9Vδ2 T-cell infusions resulted in higher OS when compared to single infusion. Future approaches are based on allogeneic γδ T-cells derived from healthy donors, either unmodified or CAR-transfected (see below) (**Table 2**).

**Table 1:** Ongoing clinical trials based on γδ T-cells.

Title	Intervention	Malignancy	Organization	Phase	Initial Date	Status	Study Identifier
**Allogeneic γδ T-cell transfer**							
TCRαβ-depleted Progenitor Cell Graft With Additional Memory T-cell DLI, Plus Selected Use of Blinatumomab, in Naive T-cell Depleted Haploidentical Donor Hematopoietic Cell Transplantation for Hematologic Malignancies	HPC-A Infusion (TCRα/β^+^ and CD19+ depleted)	ALL, AML, MDS, NK-CL, HL, NHL, JMML,CML	St. Jude Children’s Research Hospital	II	January 31, 2019	Recruiting	NCT03849651
Ex-vivo Expanded γδ T Lymphocytes in Patients With Refractory/Relapsed Acute Myeloid Leukaemia	Ex-vivo expanded allogeneic γδ T-cells from blood of related donors	AML	Wuhan Union Hospital and Jinan University, China	I	September 1, 2019	Recruiting	NCT04008381
Expanded/Activated Gamma Delta T-cell Infusion Following Hematopoietic Stem Cell Transplantation and Post-transplant Cyclophosphamide	EAGD T-cell infusion	AML,CML, ALL, MDS	University of Kansas Medical Center and In8bio Inc.	I	January 31, 2020	Recruiting	NCT03533816
Allogeneic “Gammadelta T Cells (γδ T Cells)” Cell Immunotherapy in Phase 1 Hepatocellular Carcinoma Clinical Trial	Ex-vivo expanded allogeneic γδ-T cells from related donors	HCC	Beijing 302 Hospital	I	August 15, 2020	Recruiting	NCT04518774
Gamma Delta T-cell Infusion for AML at High Risk of Relapse After Allo HCT	AlloHCT + AAPC-expanded donor T-cells	AML	H. Lee Moffitt Cancer Center and Research Institute	I/Ib	August 13, 2021	Recruiting	NCT05015426
Study of GDX012 in Patients With MRD Positive AML	GDX012. Allogeneic cell therapy enriched for Vδ1+	AML	GammaDelta Therapeutics Limited	I	August 13, 2021	Recruiting	NCT05001451
Allogeneic γδ T Cells Immunotherapy in r/r Non-Hodgkin’s Lymphoma (NHL) or Peripheral T Cell Lymphomas (PTCL) Patients	Ex-vivo expanded allogeneic γδ T-cells from related donors	NHL, PTCL	Institute of Hematology & Blood Diseases Hospital	I	January 6, 2021	Recruiting	NCT04696705
Safety and Efficiency of γδ T Cell Against Hematological Malignancies After Allo-HSCT	Ex-vivo expanded γδ T-cell infusion	AML, ALL, MDS	Chinese PLA General Hospital	I/II	September 2021	Recruiting	NCT04764513
**γδ CAR-T-cells**							
Immunotherapy With CD19 CAR γδT-cells for B-Cell Lymphoma, ALL and CLL	Allogeneic γδ CAR-T-cells (anti-CD19)	RR ALL, CLL, B-NHL	Beijing Doing Biomedical Co., Ltd.	I	October 2017	Active, not recruiting	NCT02656147
Haplo/Allogeneic NKG2DL-targeting Chimeric Antigen Receptor-grafted γδ T Cells for Relapsed or Refractory Solid Tumour	Haploidentical or allogeneic Vδ2 CAR-T-cells (anti-NKG2DL) (CTM-N2D)	RR solid tumors of different types	CytoMed Therapeutics Pte Ltd.	I	December 1, 2019	Active, not recruiting	NCT04107142
A Study of ADI-001 in B Cell Malignancies (GLEAN-1)	Lymphodepletion + ADI-001 (Anti-CD20 γδ CAR-T-cells) in monotherapy and combined with IL-2	B-NHL	Adicet Bio, Inc	I	March 4, 2021	Recruiting	NCT04735471
**Antibody-based strategies**							
First-in-Human Study of ICT01 in Patients With Advanced Cancer (EVICTION)	ICT01. monoclonal antibody targeting BTN3A	Solid Tumor, Adult Hematopoietic/Lymphoid Cancer	ImCheck Therapeutics	I/II	February 10, 2020	Recruiting	NCT04243499
Trial With LAVA-051 in Patients With Relapsed/Refractory CD1d (Cluster of Differentiation (CD)1d)-Positive CLL, MM, AML	LAVA-051. Bispecific γδ T-cell engager	CLL, AML, MM	Lava Therapeutics	I/II	July 12, 2021	Recruiting	NCT04887259
Trial of LAVA-1207 in Patients With Therapy Refractory Metastatic Castration Resistant Prostate Cancer	LAVA-1207. Bispecific γδ T-cell engager	Prostate Cancer	Lava Therapeutics	I/IIa	January 31, 2022	Recruiting	NCT05369000
**Alternative γδ T-cell-related strategies**							
Safety of TEG001 in patients with r/r AML, high-risk MDS or MM	TEG001	RR AML, high-risk MDS, MM	Gadeta B.V.	I	June 01, 2017	Recruiting	NTR6541
Novel Gamma-Delta (γδ)T Cell Therapy for Treatment of Patients With Newly Diagnosed Glioblastoma	DRI γδ T-cells modified to be resistant to TMZ + TMZ	Glioblastoma multiforme	University of Alabama at Birmingham and IN8Bio Inc.	I	February 11, 2020	Recruiting	NCT04165941
A Study to Investigate the Safety and Efficacy of TEG002 in Relapsed/Refractory Multiple Myeloma Patients	TEG002	RR MM	Gadeta B.V.	I	May 13, 2021	Recruiting	NCT04688853

AAPC, Artificial antigen presenting cell; ALL, acute lymphocytic leukemia; AlloHCT, Allogeneic hematopoietic cell transplantation; AML, Acute myeloid leukemia; B-NHL, B cell Non-Hodgkin lymphoma; CAR, Chimeric antigen receptor; CLL, Chronic lymphocytic leukemia; CML, Chronic myeloid leukaemia; DLI, Donor lymphocyte infusion; DRI, Drug resistant immunotherapy; EAGDT, Expanded/Activated γδ T-cell; HCC, Hepatocellular carcinoma; HL, Hodgkin lymphoma; HPC-A, Hematopoietic progenitor cells apheresis; HSCT, haematopoietic stem cell transplantation; JMML, Juvenile myelomonocytic leukemia; MM, Multiple myeloma; MDS, Myelodysplastic syndrome; NHL, Non-Hodgkin lymphoma; NKCL, Natural killer cell leukemia; PBMC, peripheral blood mononuclear cell; PTCL, peripheral T cell lymphoma. RR, Relapsed/Refractory; TMZ, temozolomide. Initial date, Date of first patient enrolment.

**Table 2:** Companies developing γδ T-cell-based or γδ T-cell-engaging therapies.

Organization	γδ T-cell subtype	Approach
**γδ T-cell-based therapy**		
Acepodia	information not available	Allogeneic mAb-conjugated γδ-cells
Adicet Bio	Vδ1	Allogeneic γδ CAR-T-cells
Expression Therapeutics	Vδ2	Allogeneic γδ CAR-T-cells
GammaDelta Therapeutics (acquired by Takeda)	Vδ1	Allogeneic unmodified or engineered Vδ1^+^ T-cells
Immatics	information not available	Allogeneic γδ CAR-T-cells
IN8bio (previously Incysus Therapeutics)	Vδ2	Expanded γδ T-cells engineered to achieve drug resistant immunotherapy (DRI)
Kiromic BioPharma	information not available	Allogeneic γδ CAR-T-cells genetically engineered using ABBIE non-viral gene editing technology
PersonGen BioTherapeutics	information not available	Allogeneic universal CAR (UCAR) based γδ-cells
TC BioPharm	Vδ1/Vδ2	Allogeneic unmodified γδ−cells or engineered γδ CAR-T-cells
One Chain Immunotherapeutics	Vδ1	Expanded allogeneic Vδ1^+^ T-cells for ACT
Beroni group	information not available	Allogeneic γδ ACT
**γδ T-cell-based antibody therapy**		
**Organization**	**γδ T-cell subtype**	**Approach**
Adaptate Biotherapeutics (acquired by Takeda)	Vδ1	Vδ1 bispecific T-cell engagers
ImCheck Therapeutics	Vδ2	mAbs targeting BTN isoforms to modulate γδ T-cell activation
LAVA Therapeutics	Vδ2	Vδ2 bispecific T-cell engagers
PureTech Health	Vδ1	mAb against Vδ1 to induce pro-tumoral Vδ1 T-cell killing
Shattuck Labs	Vδ2	Recombinant proteins containing heterodimeric BTN extracellular domains and a tumor targeting scFv
**Other γδ T-cell-based therapies**		
**Organization**	**γδ-T-cell subtype**	**Approach**
American Gene Technologies	Vδ2	Lentivirus to increase pAg levels in tumor cells

ACT, Adoptive cell transfer; bsTCE, bispecific T cell engager; bsVHH, bispecific Variable Heavy chain-only antibody; BTN, Butyrophilin; CAR, Chimeric antigen receptor; mAb, monoclonal antibody; pAg, phosphoantigen; scFv, Single chain variable fragment.

Application of non-Vγ9Vδ2 T-cell subsets, like Vδ1 T-cells, is of interest but lagged behind because of lack of proper expansion protocols. In 2016, Almeida *et al.* described a 3 week culture protocol based on stimulation of γδ T-cells from healthy donors or CLL patients with a combination of cytokines and anti-CD3 monoclonal antibody (mAb) clone OKT-3, resulting in 2000-fold expansion and 60-80% enrichment of Vδ1 T-cells (59). Expanded cells expressed the NK receptors NKp30 and NKp40, displayed cytotoxic activity, produced IFNγ, TNFα and no IL-17. Application of this protocol led to the development of different “delta one T” (DOT) cell products. Gamma Delta Therapeutics initiated a first-in-human phase I clinical trial in AML patients after lymphodepletion with fludarabine and cyclophosphamide (NCT05001451) (**Table 1**). This study will analyse safety and maximum tolerated dose of GDX012 and its effect on minimal residual disease, progression free survival (PFS) and OS.

#### Chimeric Antigen Receptor γδ T-Cells

Another therapeutic approach to harness the potent anti-tumor effects of γδ T-cells consists of adoptive transfer of γδ CAR-T-cells (60). CARs are chimeric antigen-recognition receptors, consisting of an ectodomain, which binds a tumor specific cell surface receptor, and endodomains, consisting of CD3ζ as the signaling domain with co-stimulatory domains to provide robust activation (*e.g.* CD28, 4-1BB, or ICOS) (61). In recent years, CAR-T-cell therapy has been extensively investigated in preclinical and clinical studies, primarily focused on conventional αβ T-cells (62–64). These autologous CAR-T-cells have triggered encouraging remission rates in patients refractory to standard treatments against, in particular, B-lymphoid malignancies. This resulted in FDA approvals of CAR-T-cell therapies for the treatment of B-cell NHL, ALL, and MM (65–69). The remarkable success of CAR-T-cell therapy revolutionized the field of adoptive cell therapy for treating hematologic malignancies and resulted in numerous ongoing clinical trials. However, CAR-T-cell therapy can be complicated by severe, potentially life-threatening, toxicities such as cytokine release syndrome (CRS), immune effector cell-associated neurotoxicity syndrome (ICANS) and other ‘on-target off-tumor’ toxicities (70). Moreover, in contrast to the results seen in hematologic malignancies, only limited antitumor effects have been obtained in patients with solid tumors.

It was hypothesized that the efficacy of CAR-T-cells could be improved and its side effects mitigated by harnessing the innate properties of γδ T-cells as a backbone for CAR. CAR-modified γδ T-cells were first described by Rischer *et al.* (71), demonstrating specific *in vitro* tumor cell lysis using ZOL-expanded Vγ9Vδ2 T-cells with CD19- or GD2-directed CARs, followed by other studies confirming these findings using γδ T-cells containing CARs against a variety of targets (72–77). Interestingly, CAR-modified Vγ9Vδ2 T-cells maintained their ability to cross-present tumor antigens to αβ T-cells *in vitro*, which may prolong the anti-tumor efficacy (76). Furthermore, γδ T-cells bearing a CD19-CAR, unlike standard CD19-αβ CAR-T-cells, had reactivity against CD19-positive and negative tumor cells *in vitro* and *in vivo*, an effect that was enhanced by ZOL (78), suggesting that CD19-directed γδ CAR-T-cells may target leukemic cells also after antigen loss and retain pAg specificity *via* their TCR. More recently, Wallet *et al.* described the generation of induced pluripotent stem cell-derived γδ CAR-T-cells (γδ CAR-iT) (79). They demonstrated sustained *in vitro* tumor cell killing by γδ CAR-iT-cells in the presence of IL-15, with markedly less IFN-γ and other inflammatory cytokines being produced compared to conventional αβ CAR-T-cells, potentially resulting in lower risk of CRS. Moreover, a single dose of γδ CAR-iT-cells resulted in potent tumor growth inhibition in a xenograft mouse model (79). **Table 2** summarizes the companies currently developing γδ CAR-T-cells.

Pre-clinical research on γδ CAR-T-cell based therapy initially focused on Vγ9Vδ2 T-cells, due to their dominant frequency in blood and their unique pAg response that allowed the specific expansion of this subset (80). Makkouk *et al.* recently showed the first example of genetically modified Vδ1 T-cells. They expanded PBMC-derived Vδ1 T-cells using an agonistic anti-Vδ1 antibody and genetically modified them to express a GPC-3 targeted CAR and to secrete IL-15 (81). In a HepG2 mouse model, these allogeneic Vδ1 CAR-T-cells primarily accumulated in the tumor and a single dose efficiently controlled tumor growth without evidence of xenogeneic GvHD. ADI-001 consists of CD20-targeting Vδ1 CAR-T-cells generated by a similar procedure by Adicet Bio (82) and is currently being used in a phase I clinical trial (NCT04735471). Recently reported interim data from this dose-escalation study showed complete responses in two and a partial response in one out of four evaluable patients already with low doses (30x10^6^ cells) of ADI-001, indicating that relatively low amounts of γδ T-cells may suffice for activity (press release). To date, no dose-limiting toxicities, GvHD, or grade 3 or higher CRS has been reported. These encouraging first results underscore the potential of Vδ1 CAR-T-cell therapy in the clinic. A complete overview of the ongoing clinical trials evaluating CAR-modified γδ T-cells is listed in **Table 1**.

### Antibody-Based Strategies

Imcheck develops ICT01, a Vγ9Vδ2 T-cell activating humanized IgG1 with a silent Fc that binds to all three BTN3A isoforms to trigger Vγ9Vδ2 T-cell activation and increased cytotoxicity against BTN3A^+^ tumor cell lines from diverse origin (21). However, this approach is not tumor specific as BTN3A is broadly expressed and could also be hampered by soluble BTN3A molecules potentially acting as decoy receptors (83). In immunodeficient NSG mice, treatment with ICT01 resulted in *in vivo* activation of adoptively transferred human Vγ9Vδ2 T-cells and delayed outgrowth of the AML cell line MOLM14 (84). The EVICTION trial is a Phase I/IIa clinical trial currently testing the effect of ICT01 in relapsed/refractory advanced-stage hematologic malignancies as a monotherapy and in a broad range of solid tumors as monotherapy or in combination with pembrolizumab (NCT04243499). Preliminary results show a good safety profile with activation of Vγ9Vδ2 T-cells and increased tumor infiltration in one melanoma patient. Stable disease has been achieved in 31% of patients treated with ICT01 as a monotherapy and in 62% in combination with pembrolizumab (84).

BsTCEs have emerged as a promising therapeutic approach for immune-oncology (85) and consist of a tumor antigen binding antibody linked to a T-cell engaging antibody fragment aiming to crosslink tumor cells and T-cells to elicit T-cell-mediated anti-tumor cytotoxicity (86, 87). Most efforts to generate bsTCEs have made use of CD3 as a T-cell engaging domain due to its role in T-cell activation. For CD3-based TCEs, proteins that are uniquely expressed or specifically overexpressed by tumor cells are the most attractive candidates for targeting, as this reduces on-target off-tumor toxicity. After approval of the CD19-CD3 bsTCE blinatumomab (88), multiple CD3-directed TCEs have been developed (89), but in many cases development has been complicated by the occurrence of adverse events such as on-target off-tumor toxicity, CRS or ICANS, highlighting the need for more tumor-selective targeting (90–92). Considering the clinical safety observed following systemic γδ T-cell activation and γδ T ACT, specific engagement of γδ T-cells using γδ bsTCEs might have an improved safety profile due to their tumor selectivity compared to CD3-bsTCEs. By avoiding detrimental co-activation of regulatory CD3^+^ T-cells observed with CD3 pan T-cell engagers (93) and their ability to bridge and engage components of both the innate and adaptive immune system, γδ bsTCEs could potentially result in increased antitumor activity.

Several γδ T-cell engaging formats are being developed and evaluated preclinically. Vγ9-TCR specific engagers directed against Her2 (94–96) and CD123 (97) were shown to cause killing of Her2 expressing cell lines and AML cell lines, respectively. The GADLEN platform (Shattuck Labs) consists of fusion proteins containing BTN heterodimers, to engage and activate Vγ9Vδ2 T-cells, bound to a tumor targeting scFv domain through an Fc linker (98). Vδ1 bsTCEs are also being developed by Adaptate Biotherapeutics. Heavy chain only antibodies occur naturally in camelids (99). Their antigen-binding fragments or variable heavy chain-only antibodies (VHH), are small, stable and with low inherent immunogenicity (100, 101). Lava Therapeutics` Gammabody™ platform combines Vδ2-specific and tumor-targeting VHHs as modules to generate bsTCE (102–105). In pre-clinical studies, Gammabody™ molecules targeting CD40, CD1d and EGFR efficiently engage Vγ9Vδ2 T-cells to kill tumor cells expressing these antigens (102–105). Two Gammabody™ molecules, are currently evaluated in clinical trials. LAVA-051, a Gammabody™ targeting CD1d is tested in a Phase I/IIa clinical trial (NCT04887259) in patients with therapy-refractory CLL, AML or MM. Preliminary data of the first 3 cohorts from this study showed a thus far good safety profile with no dose-limiting toxicities or CRS. In addition, LAVA-1207, a Gammabody™ targeting PSMA is tested in a phase I/IIa clinical trial (NCT05369000) in patients suffering from therapy-refractory metastatic castration-resistant prostate cancer. **Table 2** summarizes companies developing antibody-based γδ T-cell therapies, and **Table 1** contains clinical trials involving antibody-based γδ T-cell approaches.

### Alternative γδ T-Cell-Related Strategies

A new γδ T-cell based approach being tested in clinical trials is DeltEx drug-resistant immunotherapy (DRI). IN8Bio`s first DeltEx DRI product, INB-200, consists of expanded autologous Vγ9Vδ2 T-cells genetically modified to express a methylguanine DNA methyltransferase (MGMT). MGMT confers them resistance to temozolomide (TMZ) allowing for simultaneous treatment with TMZ and immunotherapy (106). TMZ, which is the current standard of care for glioblastoma multiforme (GBM) together with radiotherapy after resection, might sensitize tumor cells to γδ T-cell recognition through upregulation of NKG2D ligands but it also causes lymphocytopenia that is avoided by MGMT expression (107). An ongoing clinical trial (NCT04165941) is testing intracranial administration of INB-200 to the tumor site after surgical resection, followed by TMZ treatment (**Table 1**). All 4 GBM patients enrolled in this study have been reported to exceed the expected PFS for TMZ alone treatment. This technology is based on expansion and modification of autologous γδ T-cells, however, other DeltEx DRI based on allogeneic γδ T-cells (INB-400) and γδ CAR-T-cells (INB-300) are being developed.

Interestingly, although Vδ1^+^ T-cells have cytotoxic capacity, Vδ1^+^ TIL associate with poor prognosis in certain malignancies, possibly through production of IL-17 (6, 32). LYT-210 is a mAb directed towards the Vδ1^+^ TCR with the aim of eliminating these pathogenic cells (**Table 2**). Gamma-delta TCR bispecific molecules (GABs) combine the extracellular domain of the Vγ9Vδ2 TCR fused with a CD3 binding domain, allowing conventional T-cells to recognize the presence of pAg on tumor cells (108). In the presence of GABs, αβ T-cells recognized and killed the squamous cell carcinoma cell line SCC9 in a pAg dependent manner and produced increased amounts of IFNγ when exposed to patient-derived AML blasts but not with healthy hematopoietic cells indicating preferential recognition of tumor cells.

Two phase I dose-escalation clinical trials (NCT04688853; NTR6541) initiated by Gadeta are assessing the safety and tolerability of αβ T-cells engineered to express a defined Vγ9Vδ2 TCR (TEGs) in relapsed/refractory AML, MM, and high-risk myelodysplastic syndrome patients. These T-cells combine the tumor specificity of γδ T-cells with the tumor cell killing potential of αβ T-cells and show promising antitumor reactivity both *in vitro* and *in vivo*. Furthermore, chimeric PD-1 receptor (chPD1) γδ T-cells, turn PD-1 immune suppression into T-cell activation (109). The chPD1 γδ T-cells selectively killed PD-L1^+^ tumor cells in a xenograft murine model, without lysis of normal PD-L1^+^ cells or significant elevation of CRS-related cytokines. The authors reported that chPD1 γδ T-cell therapy will be assessed in a phase I/II clinical trial.

## Conclusion

Past clinical trials have demonstrated that systemic activation of Vγ9Vδ2 T-cells or adoptive transfer of autologous Vγ9Vδ2 T-cells were well tolerated and could trigger antitumor immunity. These studies have been followed by a number of trials based on Vγ9Vδ2 and the first study with Vδ1 allogeneic T-cell transfer, which would allow for donor-derived therapies. Up to this date, these trials have not resulted in major adverse effects. Most strategies that are currently under evaluation profit from the safety of γδ T-cell activation and incorporate tumor-targeting mechanisms, *e.g.* CARs or bsTCEs, which might be key to obtain more robust and consistent clinical responses. Initial results from these targeted approaches, both cell and antibody-based, show great promise and confirm the safety of Vγ9Vδ2 and Vδ1 T-cell-based strategies. However, cell-based products present challenges that are not shared by antibody-based therapies, such as high cost, difficulty of production or need of specialized facilities, and preparatory lymphodepleting chemotherapy regimens. In the near future, the results obtained by the trials described in this review will determine whether the potential of γδ T-cells can be translated into clinical benefit.

## Author Contributions

JS-E and MJ wrote the manuscript. HV co-wrote and reviewed the manuscript. LK, PP, EE, BW and TG reviewed the manuscript. All authors contributed to the article and approved the submitted version.

## Funding

The authors declare that this study received funding from LAVA therapeutics. The funder had the following involvement with the study: providing research funding to Amsterdam UMC and in designing, writing and revising the text of the mini-review.

## Conflict of Interest

JS-E, MJ and LK are funded by Lava therapeutics. HV, PP, EE, BW are employed by and hold stock of LAVA Therapeutics. TG holds stock of LAVA Therapeutics.

## Publisher’s Note

All claims expressed in this article are solely those of the authors and do not necessarily represent those of their affiliated organizations, or those of the publisher, the editors and the reviewers. Any product that may be evaluated in this article, or claim that may be made by its manufacturer, is not guaranteed or endorsed by the publisher.
